# Research progress in the use of botulinum toxin type a for post-stroke spasticity rehabilitation: a narrative review

**DOI:** 10.1080/07853890.2025.2521427

**Published:** 2025-06-23

**Authors:** Qianwen Xu, Hongbo Xiao, Zongjun Zhu, Yuanyuan Guan, Ya Wang

**Affiliations:** Anhui University of Traditional Chinese Medicine, Hefei, Anhui, China

**Keywords:** Botulinum toxin type A, stroke, spasticity, rehabilitation

## Abstract

**Background:**

Stroke is a leading cause of long-term disability and death worldwide. Spasticity after stroke seriously affects patients’ quality of life. If this state persists for a long time, it will lead to severe joint atrophy, reduced motor coordination, and even permanent disability. Therefore, clinical research has focused on the treatment of spasticity and the recovery of motor function after stroke.

**Aim:**

The aim of this paper is to explore the use of botulinum toxin type A in the rehabilitation of spasticity after stroke and to provide a theoretical basis for optimizing rehabilitation strategies, highlighting its potential value in reducing spasticity and improving motor function.

**Method:**

This article reviews the latest research progress on the application of BTX-A in spasticity after stroke, discusses the potential and challenges of BTX-A in reducing spasticity and improving motor function in patients with stroke.

**Result:**

Botulinum toxin type A (BTX-A) is a local muscle paralytic agent that has received extensive attention in recent years for its application in reducing muscle spasticity and promoting post-stroke rehabilitation.

**Conclusion:**

This article confirms that botulinum toxin type A has a significant clinical effect in treating muscle spasticity after stroke and also helps improve motor function restoration in patients. Studies have shown that botulinum toxin type A injections are effective in reducing spasticity and, when combined with rehabilitation training, can facilitate the recovery of motor function in post-stroke patients. Therefore, botulinum toxin type A has a broad application prospect in the rehabilitation of post-stroke spasticity.

## KEY MESSAGES

- The application of botulinum toxin type A (BTX-A) in post-stroke rehabilitation primarily focuses on reducing muscle spasticity and improving motor function.
- Spasticity is a common clinical manifestation of damage to the upper motor neurons. It is caused by the increased excitability of gamma motor neurons and manifests as excessive involuntary muscle contraction. Its causes include cerebral palsy, stroke, traumatic brain injury, multiple sclerosis, brain or spinal cord tumours, and spinal cord injuries

## Introduction

Movement disorders after stroke are important challenges in the rehabilitation process of patients, in which limb paralysis and spasticity seriously affect the daily life of patients, and may also cause complications such as muscle atrophy, joint stiffness and pain. Post-stroke muscle spasticity is a common complication, with an incidence rate as high as 42.6% within 1 month in the early stage, which is the difficulty of rehabilitation and the main cause of disability [1–3]. Conventional physiotherapy and medications have a role in relieving spasticity, but usually only provide temporary improvement and hardly cure the spasticity [4]. Botulinum toxin has been widely studied for its local neuromuscular blocking effects, and has unique advantages especially in reducing muscle spasticity and promoting functional recovery [5]. This study focuses on the use of botulinum toxin in stroke rehabilitation, especially its potential effects on neuroplasticity, aiming to explore its deep-rooted mechanisms in improving functional recovery and quality of life of patients.

## Methodology

The research methodology was as follows: 1. Literature search strategy: relevant studies published between January 1990 and March 2025 were collected by searching databases such as PubMed, Web of Science, Wanfang Data, and China Knowledge Network. The keywords used in the search included ‘stroke’, ‘spasticity’, ‘botulinum toxin A’, and ‘rehabilitation’. 2. Inclusion and exclusion criteria: Inclusion criteria: (1) The study subjects were patients with post-stroke spasticity; (2) The study involved the use of botulinum toxin type A and its rehabilitation effects; (3) The study design included randomised controlled trials, cohort studies, case–control studies. Exclusion criteria: (1) repeated published studies; (2) studies that did not provide specific data or results. (3) Data extraction and analysis: The extracted data were summarized and analysed, focusing on the effects of botulinum toxin type A in relieving spasticity, improving motor function, and improving quality of life. (4) Quality assessment: The Cochrane Risk of Bias Assessment Tool was used to assess the quality of the randomized controlled trials, which included the method of randomization, blinding, and completeness of the data. (5) Comprehensive analysis and discussion.

## 1. Theoretical background on botulinum toxin injections

### 1.1. Pharmacological properties

Botulinum toxin (BTX) is a bacterial exotoxin produced by Clostridium botulinum in anaerobic environments. BTX consists of a 100 KD heavy chain and a 50 strand The heavy chain of KD can specifically bind to receptors on the presynaptic membrane of nerve endings, and the light chain can hydrolyzate synaptosome-associated protein-25 (SNAP-25), thereby affecting the fusion of acetylcholine vesicles with the presynaptic membrane, inhibiting the release of acetylcholine, and causing chemical denervation such as gland secretion disorders and muscle relaxant paralysis [6–8]. BTX-A generally does not exist at measurable levels in the peripheral blood after injection. Within the recommended dose range, BTX-A diluted with normal saline is a clear liquid that is colourless to slightly yellow and free of impurities. It takes effect within three days after the injection and peaks at 1–2 weeks. The effect lasts for approximately three months each time and can be repeated as needed. The safety and side effects of BTX-A are primarily related to its mechanism of action and dosage. BTX-A should be used with caution in cases of infection at the injection site and excessive weakness or atrophy of the target muscle.

Botulinum toxin type A (BTX-A) has significant pharmacological properties for the treatment of post-stroke spasticity, mainly with the following characteristics: (1) Relieving spasticity: BTX-A effectively reduces muscle tone and alleviates muscle spasticity by blocking signal transmission at the neuromuscular junction [9]. The 2016 AAN evidence update assigns a grade A recommendation for ONA in the treatment of upper and lower limb spasticity [10]. BTX injections control spasticity and allow affected muscles to be stretched [11,12]. Studies have shown that ultrasound-guided injection of BTX-A improves the accuracy of the injection site and reduces damage to blood vessels, nerves and other tissues [13]. The new method of ultrasound-guided injection of botulinum toxin type A is a very practical and effective treatment for upper limb spasticity [14]. Although ultrasound-guided injection techniques have shown some advantages in BTX-A injections, there is no clear evidence that they are superior to EMG-guided injections. One study comparing the use of ultrasound and electromyography in BTX-A injections found that both had a role in improving function and reducing complications, but none of the techniques showed absolute superiority [15]. (2) Improvement of motor function: BTX-A injection can not only relieve spasticity but also improve the motor function of patients by reducing muscle stiffness and increasing the range of motion of the limbs [16]. Studies have shown that after BTX-A injection treatment, patients’ upper limb motor function scores, active joint range of motion, and other indicators have been significantly improved [17]. And BTX-A injections were found to improve muscle tone in the lower extremities, increase joint range of motion, and ultimately improve gait and balance in patients [18]. Thanks to the long-lasting effect and good safety profile, intramuscular injections of BoNT-A have become the first-line treatment for spasticity [19–22].

### 1.2. Mechanism discussion

#### 1.2.1. *Effects of BTX-A on neuromuscular junction* [23,24]

Botulinum toxin type A (BTX-A) exerts its effects primarily by blocking the release of acetylcholine from the neuromuscular junction. The specific mechanism of BTX-A is as follows. First, BTX-A binds to specific receptors in nerve terminals and enters cells through receptor-mediated endocytosis. Intracellular transport: Upon entry into the cell, the light chain of BTX-A is released into the cytoplasm to cleave SNAP-25. Inhibition of neurotransmitter release: SNAP-25 is an essential protein in the presynaptic membranes of nerve terminals, which is responsible for the release of neurotransmitters (acetylcholine) from vesicles into the synaptic cleft. After cleavage of SNAP-25 by BTX-A, the release of acetylcholine is blocked, resulting in muscle contraction dysfunction. (4) The long-term impact of the neuromuscular joint: this blocking role within 2-3 days after injection in clinical manifestations, 5-6 weeks after the role of peak and general functions can be recovered within 12 weeks after injection, because nerve endings ‘sprout’ and end plate to form new connections.

#### 1.2.2. *Potential effects of BTX-A on nerve regeneration and repair after stroke* [25]

Although BTX-A is mainly used for the treatment of muscle spasticity and cosmetology, its potential role in nerve regeneration and repair after stroke cannot be ignored: (1) Nerve regeneration: BTX-A may promote the regeneration and repair of damaged nerves by reducing muscle spasticity and improving local blood flow. (2) Neuroprotection: BTX-A may have a neuroprotective effect, protecting damaged nerve cells by reducing nerve over-excitation and oxidative stress. Rojewska’s review highlights that The neuroprotective effects of botulinum toxin type A are mainly in the inhibition of neuroinflammation, modulation of glial cell function, and relief of neuropathic pain. By reducing neuroinflammation, botulinum toxin type A may provide a more favourable environment for nerve repair [26]. (3) Clinical research: Although there is a lack of large-scale clinical trial data, some preliminary studies have suggested that BTX-A may have a positive effect on the rehabilitation of patients after stroke.

#### 1.2.3. *Role of BTX-A in neuroplasticity* [27,28]

Neuroplasticity refers to the ability of the nervous system to adapt to environmental changes through structural and functional changes during development and maturation as well as after injury. The role of BTX-A in neuroplasticity is mainly reflected in the following aspects: (1) Synaptic plasticity: BTX-A may affect the plasticity of neural synapses and change the transmission and processing of neural signals by blocking the release of acetylcholine [29]. A study by Kerzoncuf et al. examined the effects of botulinum toxin type A on synaptic plasticity at Ia-motor neuron synapses in patients with hemiparesis after stroke. It was found that BoNT/A treatment not only relieves spasticity through its peripheral effects (e.g. muscle relaxation), but may also improve motor function by modulating synaptic transmission in the central nervous system [30]. (2) Neural network remodelling: In some cases, BTX-A may promote the remodelling of damaged neural networks to help restore function. Delcamp’s findings are the first to provide strong preliminary evidence of a modification of β-CMC in stroke patients during an active movement at short and long term after a treatment combining BoNT-A injections and rehabilitation [31]. (3) Learning and memory: BTX-A may affect certain neural pathways related to learning and memory, although research on this topic is still in its infancy. The review by Hok et al. provides a comprehensive overview of electrophysiological and functional imaging evidence of the effects of botulinum neurotoxin (BoNT) on the brain, furthering our understanding of the pathophysiology of dystonia and spasticity and thus contributing to the development of novel therapeutic strategies based on neuroplasticity [32–34].

Although BTX-A is able to modulate synaptic transmission and influence long-duration potentiation, thus providing a potential physiological basis for neuroplasticity, these mechanisms are not sufficient by themselves to achieve functional recovery. Neuroplasticity performance and functional improvement need to be achieved through active training and learning.

### 1.3. BTX-A: dose, timing, and product efficacy

#### 1.3.1. Study on the use of different doses of BTX-A

The study by Facciorusso et al. states that the treatment of spasticity with botulinum toxin involves specific dosage guidelines that vary between formulations [35]. Some studies have examined the relationship between BTX-A dose and spasticity improvement, but the results suggest that dose is not the only influencing factor [36]. Current evidence from the literature suggests that higher doses of BoNT-A are effective in reducing spasticity of upper and lower limbs after stroke, with rare occurrence of mild adverse effects [37]. Zorowitz’s study provides clinicians with practical data on the use of BoNT-A in real practice for the treatment of lower limb spasticity, which can help to optimise treatment regimens and dosage adjustments [38]^.^ Patel et al. investigated that DaxibotulinumtoxinA injection demonstrated favourable efficacy and safety in the treatment of upper limb spasticity in adults following stroke or traumatic brain injury. This study supports DAXI as an effective therapeutic option, especially at higher doses, to significantly improve patients’ symptoms of upper limb spasticity [39]^.^ Guo Aihong et al. explored the effects of different doses of botulinum toxin A injection on muscle strength and walking function in patients with post-stroke muscle spasticity. The results show that botulinum toxin type A can promote the recovery of muscle strength and walking function in patients with post-stroke muscle spasticity, and improve the ability of daily living, and 400 U is the best injection dose. The high-dose group had the fastest onset time, longest duration, and best effect, while adverse reactions were not significantly increased, which was the best dose [40].

#### 1.3.2. Studies on the use of BTX-A in different periods

There is no consensus on the ideal time of botulinum toxin type A administration for the treatment of lower-limb spasticity in stroke patients. Early injection may weaken limb strength and affect gait recovery, while late injection may be irreversible due to contracture. Lower limb spasticity, especially ankle plantar flexor spasticity, can lead to asymmetric weight-bearing and inefficient gait patterns during walking [41]. Rosales’s meta-analysis aimed to evaluate the effects of early BoNT-A injection for post-stroke spasticity on improvements in hypertonicity, disability, function and associated pain. The literature search yielded six studies reporting the effects of BoNT-A treatment within 3 months post-stroke. These results demonstrate the beneficial effects of BoNT-A treatment on improving hypertonicity within 3 months post-stroke and emphasize the importance of concomitant neurorehabilitation therapy [42]. Ma Shanxin et al. showed that botulinum toxin type A treatment consistently improved spasticity regardless of the duration of stroke onset, and none of the patients’ gait speed slowed or worsened after treatment. The effect was particularly pronounced in patients who received botulinum toxin type A treatment early (within 6 months) [43].

#### 1.3.3. Efficacy and formulation differences in type a botulinum toxin products

Botulinum toxin is a bacterial exotoxin produced during the proliferation of Clostridium botulinum, which belongs to the neurotoxin. Among them, type A BT (BT/A), because of its effect on neuromuscular connection strong affinity and for a long time [44], is known as the earliest most widely applied in clinical and research of BT type. BT/A can have cholinergic nerve endings with presynaptic membrane receptor specificity, blocking c2-style protein complexes and inhibiting presynaptic membrane acetylcholine release, causing chemical denervation of muscle, leading to muscle relaxation [45]. According to its antigenicity, botulinum toxin is divided into A, B, C, D, E, F, and G, and type A BT has been widely used in clinical treatment, becoming a focal convulsion and spasticity in the treatment of diseases. Domestic approved type A BT are on a botulinum toxin A (ONA) and BTX-a. With the increasing clinical application of ONA and BTX-A in China, the differences in therapeutic efficacy between these two drugs have attracted increasing attention. The BT preparation is mainly composed of three parts: botulinum neurotoxin (BoNT), complexing protein (CP), and vehicle [46], among which only botulinum neurotoxin has a therapeutic effect. ONA and BTX-A are both type A BT, so there is no difference in the components of botulinum neurotoxin. The CP of ONA and BTX-A was composed of large aggregates of hemagglutinin-related proteins and non-toxic non-hemagglutinin-related proteins. In terms of vehicle, ONA contains human serum albumin and sodium chloride, whereas BTX-A contains bovine gelatin protein, dextrose, and sucrose [21].

## 2. BTX-A in the treatment of post-stroke spasticity

### 2.1. BTX-A for lower limb spasticity in stroke patients

Stroke is a common neurological disease, and muscle spasticity is one of the most common sequelae after stroke. The incidence of muscle spasticity has been reported to be as high as 42.6% within 6 months after stroke [47], and the prevalence of muscle spasticity is 25%–43% within 1 year after stroke onset, which seriously affects the walking ability and daily life self-care ability of patients [48,49]. Years of practice by domestic and foreign experts [50,51] have proven that botulinum toxin type A (BTX-A) is safe and effective in the treatment of stroke limb spasticity. In 2016, the American Rehabilitation Guidelines [52]proposed that the targeted injection of botulinum toxin into the lower limb muscles to reduce spasticity and gait abnormalities was regarded as level 1 recommendation level A evidence. Lower limb spasticity after stroke can affect the balance and walking function of patients and seriously affect the ability of daily living of patients, which has become a bottleneck problem to be solved urgently in stroke rehabilitation [53]. Wang et al. showed that botulinum toxin type A injection combined with spasmodic electromyographic stimulation can improve the efficacy of post-stroke lower limb muscle spasticity, alleviate the degree of muscle spasticity in the lower limbs, promote the restoration of the affected limb’s muscle structure and function, and improve the patient’s motor ability and quality of life [54]. Reebye et al. highlight the proven efficacy of botulinum toxin in spasticity treatment, but its impact on functional recovery may be enhanced when combined with a rehabilitation programme [55].

### 2.2. BTX-A for upper limb and gait-related spasticity in stroke patients

Stroke is the leading cause of focal or segmental limb spasticity as well as hemiplegia in elderly patients. Post-stroke spasticity, which can arise from either ischemic or haemorrhagic cerebrovascular insults, is often mixed and variable with several components of hyperkinetic movements [56]. Foley N et al. showed that the use of BTX-A was associated with moderate improvement in upper-extremity activity capacity or performance after stroke [57]. A review by Varvarousis et al. showed that botulinum toxin injections were effective in reducing lower limb hypertonia in patients with post-stroke hemiparesis, while BTA injections reduced spasticity and improved gait indices in post-stroke patients [58]. The study by Stoquart et al. states that BTX-A rectus femoris injection may be beneficial in patients with a stiff-knee gait after stroke, particularly in patients with some knee flexion (>10 degrees) [59]. Cofré Lizama et al. study noted that the effects of BoNT on walking after stroke will depend on the muscles compromised by spasticity and the muscles injected. And gait analysis can be used for better BoNT clinical decision making in stroke patients [60]^.^ Cotinat et al. point out, based on the current findings, that spasticity treatment focusing only on the plantarflexors does not appear to be an effective means of improving gait after stroke, and argue that the optimal approach to improving gait must be multidimensional [61].

## 3. BTX-A combined with other rehabilitation therapies in spasticity

### 3.1. General advantages of combining BTX-A with rehabilitation therapies

BTX-A in combination with other rehabilitation therapies has unique advantages and features in the treatment of post-stroke spasticity. (1) Significant efficacy: BTX-A injection significantly reduced muscle tension and relieved muscle spasticity. (2) The auxiliary effect of comprehensive rehabilitation treatment: BTX-A injection is usually used in combination with other rehabilitation treatments, such as physical therapy and exercise therapy, to improve the therapeutic effect. In stroke patients, BTX-A injection combined with rehabilitation training, such as stretching and functional exercise training, can improve motor function and reduce muscle tension. (3) Safety and side effects: BTX-A injections have relatively fewer side effects. Common adverse effects include injection site pain and transient muscle weakness; however, these reactions usually recover within 2–4 weeks.

### 3.2. Specific applications of BTX-A in combination with rehabilitation treatments

(1) Neuromuscular Electrical Stimulation: NMES has been proven effective in enhancing functional performance, coordination, and muscle strength [62,63] and the technique is increasingly being employed as an adjuvant treatment to enhance the efficacy of BTX injections [64]. And the use of NMES alongside physical exercises can enhance the effects of BTX injections [65]. (2) Ultrasound-Guided Injections: Ultrasound-guided botulinum toxin type A injections combined with rehabilitation programs decrease spasticity and improve the upper extremity motor functions in stroke patients [14]. (3) Focal Extracorporeal Shock Wave Therapy: Cumulative evidence from clinical randomised controlled trial studies suggests that combined and simultaneous treatment with botulinum toxin and focal extracorporeal shock wave reduced spasticity in a more effective and prolonged way than treatment with botulinum toxin only [18,66]. (4) Hybrid Assistive Limb: Combining single-joint hybrid assistive limb (HAL-SJ) with botulinum toxin A (BTX-A) therapy is novel and has great therapeutic potential for the rehabilitation of stroke patients with upper limb paralysis [67]. (5) Standing Training and Exercise Programmes: The study by Liu J et al. demonstrated that botulinum toxin type A combined with standing training and an exercise program could significantly relieve lower limb muscle spasticity in stroke patients and improve the motor function and balance ability of the affected limb [68]. (6) Repetitive Transcranial Magnetic Stimulation (rTMS): Shuqin et al. [69]confirmed that BTX-A combined with repetitive transcranial magnetic stimulation (rTMS) could significantly improve upper-limb spasticity in patients after stroke. (7) Intermittent Theta Burst Stimulation (i-TBS): Du Junqiu et al. [17]confirmed that intermittent θ rhythm stimulation (i-TBS) combined with botulinum toxin type A could significantly improve the motor function of the upper limb in hemiplegia and improve the ability of daily living and quality of life, which is worthy of clinical application. (8) Musculoskeletal Ultrasound and Electromyography: Lin Qingyang et al. [70] confirmed that BTX-A injection guided by musculoskeletal ultrasound combined with electromyography in the treatment of foot drop after stroke could effectively improve the range of motion of the ankle joint and walking ability.

Although BTX-A plays an important role in improving muscle spasticity and facilitating the recovery of motor function, the primary role of BTX-A is to create more favorable conditions for rehabilitation rather than to independently improve motor control. It is not a primary treatment in itself, but rather an adjunct to create optimal conditions for motor training and learning. Data from several studies have shown that decreasing muscle tone does make it easier for antagonist muscles to move into a motor state, but this effect needs to be combined with rehabilitation training to translate into actual functional improvement.

## 4. Discussion and summary

The application of botulinum toxin type A (BTX-A) in post-stroke rehabilitation primarily focuses on reducing muscle spasticity and improving motor function. BTX-A blocks acetylcholine release from the neuromuscular junction and inhibits muscle contraction, thereby reducing spasticity and improving motor function. Although BTX-A has shown some potential in post-stroke rehabilitation, it also faces some challenges: (1) The optimal timing of BTX-A intervention: The optimal timing of BTX-A intervention in post-stroke rehabilitation is still unclear. Some studies have suggested that early rehabilitation interventions may be more effective, but more research is needed to determine the specific timing and methods. (2) Duration and intensity of rehabilitation therapy: The duration and intensity of rehabilitation therapy have an impact on the recovery from stroke, but there is a lack of clear guidance. Systematic reviews have shown that higher treatment doses do not necessarily lead to better recovery. (3) Application of new technologies and devices: Although some new technologies and devices, such as weight-support stepping machines and robot-assisted technology, have been used in stroke rehabilitation, their effects require further verification. (4) Challenges in animal experiments: Many human post-stroke dysfunctions are difficult to simulate in animal models, which limits the research progress on the application of BTX-A in stroke rehabilitation.

The efficacy and safety of botulinum toxin (BoNT-A) in the treatment of spasticity has been widely recognized [71]. However, despite its demonstrated significant benefits in improving patients’ symptoms and quality of life, some challenges remain in its long-term application. For example, repeated injections over a long period of time may lead to diminished efficacy, which may be related to factors such as drug dosage, injection intervals, and individual patient differences. Reducing spasticity itself does not necessarily translate directly into improved motor function. Many studies assessing the effects of BTX-A have often overlooked its impact on the quality of training. For example, one study found that while muscle spasticity was significantly reduced after BTX-A injections, the improvement in motor function was not significant until combined with rehabilitation training [72]. In addition, optimising treatment strategies to further improve the long-term efficacy of botulinum toxin and patient compliance remains a focus of current research!

## Data Availability

Data availability is not applicable to this article, as no new data were created or analyzed in this study.
